# Paraneoplastic Motor Neuron Disease in a Patient With Sigmoid Colon Adenocarcinoma: A Case Report

**DOI:** 10.7759/cureus.67865

**Published:** 2024-08-26

**Authors:** Keesari Sai Sandeep Reddy, Gunasekaran Nallusamy, Priyadarshini Varadaraj, Vivekanandan T, Subbiah SenthilNathan

**Affiliations:** 1 Internal Medicine, Saveetha Medical College and Hospital, Saveetha Institute of Medical and Technical Sciences, Saveetha University, Chennai, IND

**Keywords:** type 2 diabetes mellitus, neuropathy, autoimmune response, sigmoid colon adenocarcinoma, motor neuron disease, paraneoplastic neurological syndromes

## Abstract

Paraneoplastic neurological syndromes (PNS) are a rare and diverse group of disorders caused by immune-mediated effects of malignancies. These syndromes are very rare and often present diagnostic and therapeutic challenges. Motor neuron disease as a paraneoplastic condition is particularly uncommon, especially in association with gastrointestinal malignancies like sigmoid colon adenocarcinoma.

A 62-year-old male with type 2 diabetes mellitus (T2DM) presented with chronic diarrhea and a three-year history of progressive bilateral limb weakness. Initial symptoms were attributed to diabetic neuropathy, but the rapid progression and severity warranted further investigation. Neurological examination revealed hypotonia, muscle wasting, and absent reflexes in all four limbs. Diagnostic tests, including electromyography (EMG) and nerve conduction studies, confirmed motor sensory axonal neuropathy. A colonoscopy revealed a mass in the sigmoid colon, and a biopsy confirmed adenocarcinoma. The patient was managed with surgical resection of the tumor, adjuvant chemotherapy, and immunomodulatory treatments, resulting in the stabilization of neurological symptoms.

This case highlights the importance of considering paraneoplastic syndromes in patients with unexplained neurological symptoms, particularly when a malignancy is suspected or known. Early recognition and a multidisciplinary approach are crucial for improving patient outcomes. Further research is needed to understand the pathophysiological mechanisms and develop sensitive biomarkers for early detection.

## Introduction

Paraneoplastic neurological syndromes (PNS) represent a heterogeneous group of disorders caused by immune-mediated effects of malignancies rather than direct tumor invasion, metastasis, or compression. These syndromes, affecting less than 1% of all cancer patients, pose significant diagnostic and therapeutic challenges due to their rarity and the broad spectrum of clinical manifestations [1]. PNS can affect any part of the nervous system, with manifestations potentially involving the central, peripheral, or autonomic nervous systems. Notably, these neurological symptoms can precede the diagnosis of the underlying malignancy, often leading to delays in recognition and management [2,3].

The clinical presentation of paraneoplastic motor neuron disease can mimic that of more common neurodegenerative diseases, making the differential diagnosis challenging. Symptoms typically include progressive weakness, muscle wasting, and fasciculations, which are not confined to the distribution of a single nerve or root, distinguishing them from more localized neurological disorders [4]. The association between motor neuron disease and malignancies like sigmoid colon adenocarcinoma is particularly rare, and literature documenting such cases is sparse. This rarity, combined with the non-specific nature of early symptoms, can complicate timely diagnosis and management [5].

The significance of this case lies in its contribution to the limited body of knowledge on paraneoplastic motor neuron disease associated with gastrointestinal malignancies. Given the complexity and the potential for significant impact on patient management, this case underscores the necessity of a multidisciplinary approach involving neurologists, oncologists, and other specialists. Furthermore, it highlights the critical importance of considering PNS in differential diagnosis when patients present with unexplained neurological symptoms and a known or suspected malignancy [6,7].

This case report seeks to emphasize the importance of considering PNS in patients presenting with unexplained neurological symptoms, particularly when a malignancy is known or suspected. It also aims to shed light on the need for early recognition and a multidisciplinary approach to improve patient outcomes.

## Case presentation

A 62-year-old male with a longstanding history of type 2 diabetes mellitus (T2DM) presented to our neurology clinic with a perplexing combination of chronic diarrhea and progressive bilateral limb weakness. Over the past three years, his life had been marred by a steady decline in mobility, significantly impeding his daily activities. Initially, his symptoms were attributed to diabetic neuropathy, a common complication of T2DM. However, the relentless progression of his weakness and other symptoms demanded a deeper investigation.

The patient reported experiencing chronic diarrhea for the past two months, characterized by 1-2 watery, non-bloody, non-foul-smelling episodes per day. This gastrointestinal distress was accompanied by a three-year history of progressive weakness affecting both his upper and lower limbs. The weakness began insidiously in his lower limbs and gradually spread to his upper limbs, significantly impacting his ability to perform routine tasks. Over the past year, he also experienced mild, non-specific abdominal pain, discomfort, involuntary facial twitching, and a burning sensation with numbness in his lower limbs.

His medical history was notable for T2DM, which he managed with oral hypoglycemic agents. There was no history of hypertension, tuberculosis, or asthma. He had undergone surgical management for a diabetic foot ulcer and had a history of occasional alcohol consumption, which he had ceased three years earlier. His diet was mixed, his sleep patterns were normal, and he reported altered bowel habits due to his current complaint.

**Figure 1:**
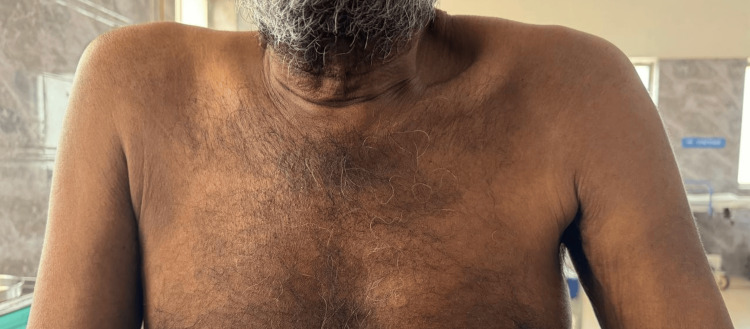
Generalised muscle wasting

**Figure 2:**
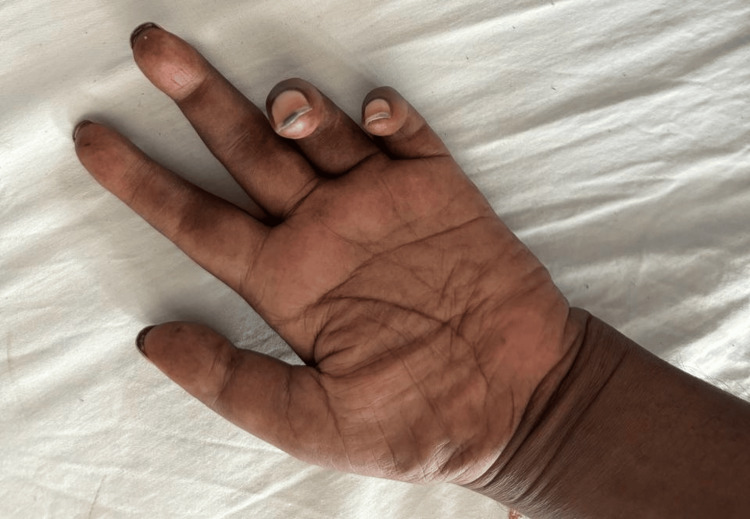
Muscle wasting in hand

On physical examination, the patient appeared alert and oriented, with stable vital signs: temperature 98.6°F, pulse 85 bpm, blood pressure 110/70 mmHg, respiratory rate 16 breaths per minute, and oxygen saturation of 99% on room air. Neurological examination revealed pronounced hypotonia in all four limbs, accompanied by visible muscle wasting in both upper and lower limbs (Figures 1-2). Muscle strength was graded as 4/5 in the upper limb flexors, 2/5 in the upper limb extensors, 2/5 in the lower limb flexors, and 4/5 in the lower limb extensors. Deep tendon and plantar reflexes were absent. Sensory examination demonstrated a glove-and-stocking pattern of sensory loss, more pronounced in the lower limbs, involving both small and large fibers.

Investigations

**Figure 3:**
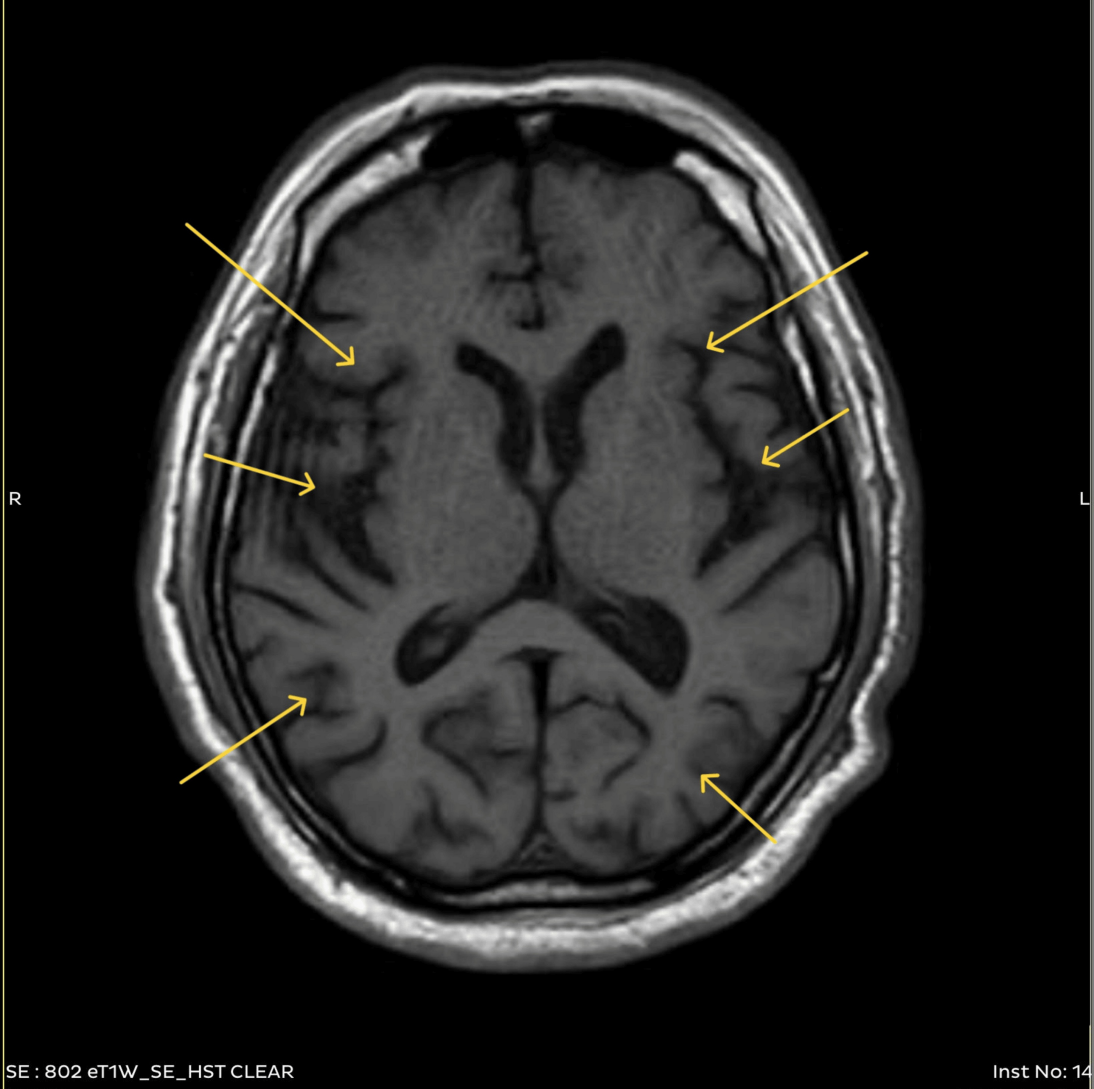
T1W MRI of axial section of the brain showing diffuse brain parenchymal atrophy as evidenced by prominent gyri and sulci T1W MRI: T1-weighted magnetic resonance imaging

**Figure 4:**
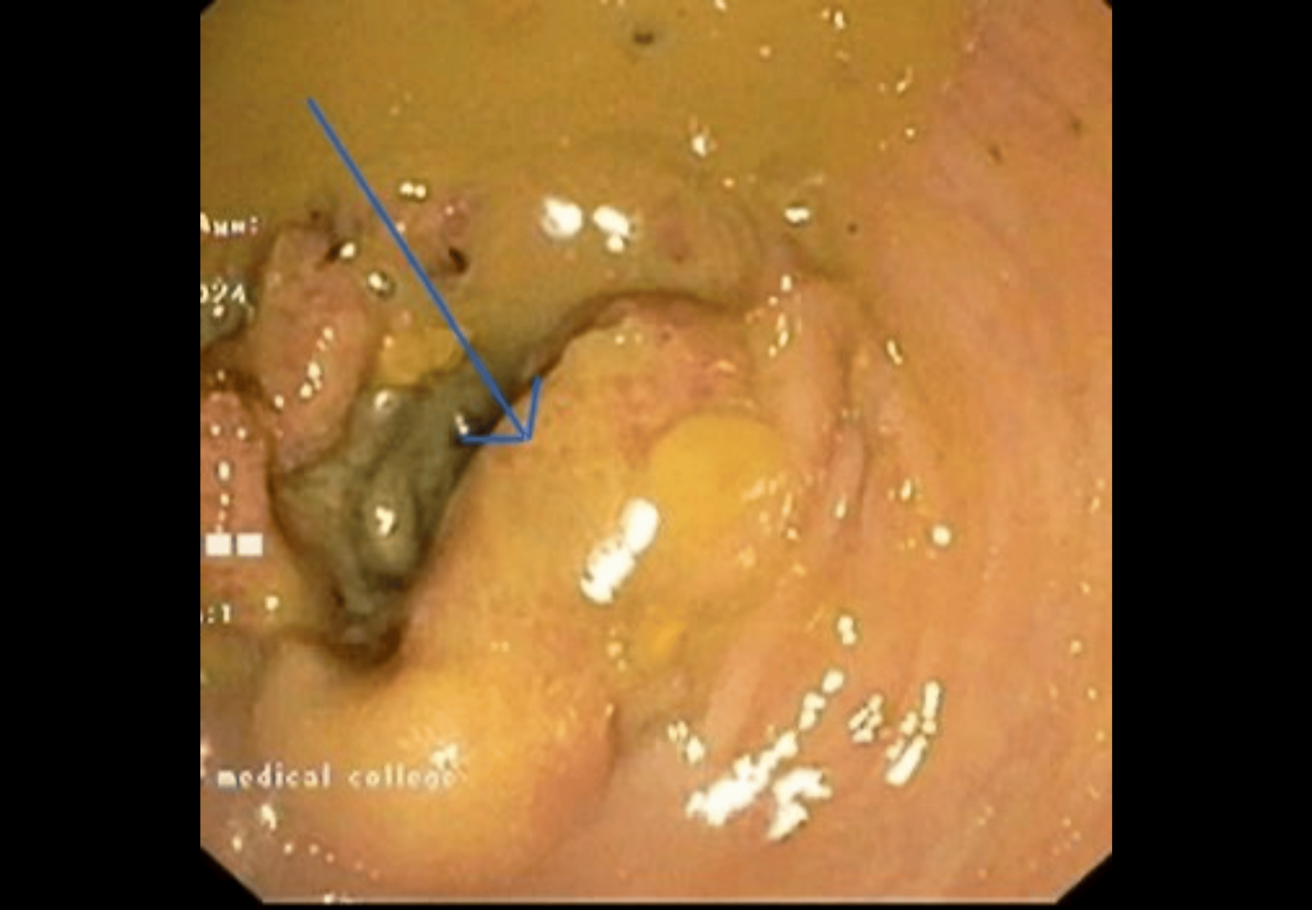
Colonoscopy showing a mass in the sigmoid colon

The patient underwent a comprehensive series of diagnostic tests to unravel the cause of his symptoms. As mentioned in Table 1, a complete blood count (CBC) revealed microcytic hypochromic anemia and leukocytosis. Electrolytes and renal function tests were within normal limits. Liver function tests showed a mild elevation in alkaline phosphatase (ALP). Glycated hemoglobin (HbA1c) was elevated at 9.90%, indicating poorly controlled diabetes. Fasting blood sugar was 190 mg/dL. Magnetic resonance imaging (MRI) of the brain and spine revealed cervical spinal cord thinning and diffuse brain atrophy, suggestive of chronic ischemic changes and myelopathy (Figure 3). Electromyography (EMG) and nerve conduction studies confirmed motor sensory axonal neuropathy, suggestive of motor neuron disease. A colonoscopy unveiled a mass in the sigmoid colon, and a biopsy confirmed adenocarcinoma (Figure 4). The stool occult blood test was positive, indicating gastrointestinal bleeding.

**Table 1:** Investigations and interpretations CBC: Complete blood count; TLC: Total leukocyte count; ESR: Erythrocyte sedimentation rate; HbA1c: Glycated hemoglobin; ALP: Alkaline phosphatase; MRI: Magnetic resonance imaging; EMG: Electromyography

Investigation	Results	Interpretation
CBC	Hb: 9.7 g/dL, TLC: 12690 /μL, Platelets: 7.51 L/μL, ESR: 120mm/hr	Microcytic hypochromic anemia and leukocytosis. Indicative of possible chronic disease and inflammation
Electrolytes	Within normal limits	Normal. No electrolyte imbalance contributing to symptoms
Renal function tests	Within normal limits	Normal. Renal function is not a contributing factor
Liver function tests	ALP: 103 U/L	Mild elevation. Possible liver involvement but not significant
HbA1c	9.90%	Confirms chronic hyperglycemia
Fasting blood sugar	190 mg/dL	Consistent with poorly controlled diabetes
MRI brain and spine	Cervical spinal cord thinning, diffuse brain atrophy	Suggestive of chronic ischemic changes and myelopathy. Underlying neurological issues linked to symptoms
EMG and nerve conduction study	Motor sensory axonal neuropathy	Suggestive of motor neuron disease. Confirms the presence of severe neurological impairment
Colonoscopy	Mass in the sigmoid colon	Biopsy confirmed adenocarcinoma. Identifies primary malignancy contributing to paraneoplastic syndrome
Stool occult blood test	Positive	Suggestive of gastrointestinal bleeding

Differential diagnosis

The patient presented with a combination of chronic diarrhea and progressive bilateral limb weakness, which necessitated a thorough differential diagnosis to determine the underlying cause of his symptoms. Several potential diagnoses were considered:

Diabetic neuropathy: Given the patient's long history of poorly controlled T2DM with an elevated HbA1c level, diabetic neuropathy was initially suspected. Diabetic neuropathy typically presents with distal symmetric polyneuropathy, characterized by sensory deficits, pain, and autonomic dysfunction. However, the rapid progression of motor weakness and significant muscle wasting observed in this patient was atypical for diabetic neuropathy alone, suggesting an additional or alternative pathological process.

Amyotrophic lateral sclerosis (ALS): ALS is a progressive neurodegenerative disease affecting motor neurons in the brain and spinal cord, leading to muscle weakness, atrophy, and fasciculations. The absence of upper motor neuron signs and the presence of sensory involvement made ALS less likely in this patient. Although it remained a consideration due to the severe motor symptoms, it was less likely as an MRI brain/spine showed no features suggesting the condition.

Chronic inflammatory demyelinating polyneuropathy (CIDP): CIDP is an immune-mediated disorder characterized by progressive weakness and sensory dysfunction. It typically presents with both motor and sensory deficits, and nerve conduction studies often show demyelination. The motor sensory axonal neuropathy observed in this patient's EMG and nerve conduction studies was not consistent with CIDP, which usually shows demyelinating features.

Vitamin deficiencies: Deficiencies in vitamins such as B6, B12, and E can cause peripheral neuropathy and neurological symptoms. The patient's nutritional status and laboratory evaluations did not support vitamin deficiency as a primary cause.

Paraneoplastic neuropathy: Paraneoplastic syndromes are immune-mediated responses to cancer that can affect the nervous system. The patient's chronic diarrhea and progressive weakness, coupled with the detection of a sigmoid colon mass, raised the suspicion of a paraneoplastic etiology. Paraneoplastic neuropathies can present with a wide range of neurological symptoms, and the presence of adenocarcinoma confirmed by biopsy further supported this diagnosis.

Given the combination of progressive neurological symptoms and the discovery of a sigmoid colon adenocarcinoma, paraneoplastic motor neuron disease secondary to the malignancy was strongly suspected. This diagnosis was supported by the temporal relationship between the onset of neurological symptoms and the detection of the tumor, as well as the exclusion of other potential causes.

Management

The patient was managed with a multidisciplinary approach involving neurology, oncology, and gastroenterology. Treatment included surgical resection of the sigmoid tumor, initiation of adjuvant chemotherapy, and supportive care for neurological symptoms, including high-dose corticosteroids.

Outcome

Postoperative recovery was uneventful, and the patient showed some stabilization of neurological symptoms, although significant recovery was limited due to the chronic nature of the neurological damage.

## Discussion

This case report illustrates the complexities and diagnostic challenges associated with paraneoplastic motor neuron disease in a patient with sigmoid colon adenocarcinoma and T2DM. The patient’s progressive bilateral limb weakness, initially attributed to diabetic neuropathy, highlights the diagnostic complexity when common conditions mask more severe underlying pathologies.

Motor neuron diseases, particularly in the context of paraneoplastic syndromes, are uncommon and often misdiagnosed due to their overlapping symptoms with other neurological disorders. In this case, the rapid progression of muscle wasting and weakness, coupled with the absence of upper motor neuron signs, raised suspicion for an alternative diagnosis beyond diabetic neuropathy. EMG and nerve conduction studies confirmed motor sensory axonal neuropathy, which aligned with the presentation of paraneoplastic motor neuron disease [8,9].

The discovery of a sigmoid colon mass during the diagnostic workup was pivotal. The biopsy confirmed adenocarcinoma, providing a crucial link to the neurological symptoms. Paraneoplastic neurological syndromes are immune-mediated and can precede the detection of the underlying malignancy, as seen in this patient. The immune system’s response to the tumor antigens likely cross-reacted with neuronal antigens, leading to neuronal degeneration and motor neuron disease [10,11].

The management of this patient underscores the importance of a multidisciplinary approach. Surgical resection of the sigmoid tumor, combined with adjuvant chemotherapy and immunomodulatory treatments, aimed to reduce the tumor burden and mitigate the immune response causing neuronal damage. Despite these interventions, significant neurological recovery was limited, reflecting the chronic and often irreversible nature of paraneoplastic motor neuron disease [12,13].

The coexistence of T2DM further complicated the clinical picture. Diabetic neuropathy, a common complication of diabetes, typically presents with distal symmetric polyneuropathy, characterized by sensory deficits, pain, and autonomic dysfunction. In this patient, the burning sensation and numbness in the lower limbs were consistent with diabetic neuropathy. However, the progressive motor weakness and muscle wasting were atypical for diabetic neuropathy alone, indicating an additional pathological process. This overlap of symptoms can delay the diagnosis of paraneoplastic syndromes, as seen in this case [14,15].

The temporal relationship between the onset of neurological symptoms and the diagnosis of sigmoid colon adenocarcinoma, coupled with the exclusion of other potential causes, strongly suggested a paraneoplastic etiology. This case underscores the importance of considering paraneoplastic syndromes in patients with known malignancies presenting with new, unexplained neurological symptoms. Early recognition and prompt intervention are crucial for improving outcomes. Clinicians should maintain a high index of suspicion for paraneoplastic syndromes in similar clinical scenarios, ensuring comprehensive diagnostic evaluations are undertaken [16,17].

Further research is necessary to better understand the pathophysiological mechanisms linking colorectal cancer to paraneoplastic motor neuron disease. Developing sensitive biomarkers for early detection could significantly improve prognosis by enabling earlier and more targeted interventions. Additionally, studies exploring the effectiveness of various immunomodulatory treatments in paraneoplastic motor neuron disease could provide valuable insights into optimizing patient management [18].

## Conclusions

In conclusion, paraneoplastic lower motor neuron disease, particularly in association with colorectal cancer, remains a rare and challenging diagnosis. This case underscores the critical role of a comprehensive, multidisciplinary approach in diagnosing and managing such complex presentations. Early recognition and targeted treatment of both the malignancy and the neurological syndrome are essential to improve patient outcomes and quality of life. Clinicians should remain vigilant for paraneoplastic syndromes in patients with unexplained neurological symptoms, ensuring a broad and thorough differential diagnosis to guide appropriate management strategies.
